# Microscopic Differentiation of Plasmonic Nanoparticles for the Ratiometric Read-out of Target DNA

**DOI:** 10.1038/s41598-017-15256-1

**Published:** 2017-11-07

**Authors:** Zhenjie Wu, Rui Yang, Di Zu, Shuqing Sun

**Affiliations:** 1 Institute of Optical Imaging and Sensing, Shenzhen Key Laboratory for Minimal Invasive Medical Technologies, Graduate School at Shenzhen, Tsinghua University, Shenzhen, 518055 People’s Republic of China; 2 Department of Physics, Tsinghua University, Beijing, 100084 People’s Republic of China

## Abstract

The development of highly sensitive and rapid methods for detecting DNA is of critical importance. Here, we describe a strategy for the digital detection of target DNA at the femto-molar level. Individual DNA molecules were encoded with a single gold nanorod (AuNR), separated and enriched by magnetic immune-separation. The coding gold nanorods were then de-hybridized and dispersed into a gold nanosphere (AuNS) solution at a certain concentration, and both gold nanoparticles were immobilized on glass slides for dark-field microscopic imaging. Using an in-house Matlab program, the concentration of the target DNA was calculated based on the ratio of the coding gold nanorods to gold nanospheres. By combining the coding of individual biomolecules with a single gold nanorod and the use of gold nanospheres as an internal standard, a method for the rapid and accurate digital detection of target DNA to the femto-molar level was developed.

## Introduction

Currently, immunoassay and nucleic acid hybridization are the most widely used bioassays and play very important roles in clinical diagnosis, food and environmental analyses, and biological and biomedical studies^1–3^. The development of highly sensitive and rapid methods for detecting DNA is of critical importance^4,5^. However, the complexities of the experimental procedures and high costs have greatly limited their application on a broad scale. Currently, noble metal nanoparticles, such as gold nanospheres (AuNSs), silver nanospheres (AgNSs), and gold nanorods (AuNRs), have been widely used for bioanalysis and diagnostics as optical transducers because of their unique and superior optical properties^6–9^. Actually, accurate, sensitive, rapid, and low-cost quantification of gold nanomaterials is desirable for chemical and biological sensing as well as clinical diagnostics^10,11^. Recently, a single-nanoparticle (NP) counting technique was reported. Based on this method, target DNA at low concentrations can be detected by counting the encoding nanoparticles^12^. A detection limit to atto-molar level for DNA has been achieved through enrichment and absolute enumeration of the coding nanoparticles without signal amplification^12^. Although this is a promising and robust strategy, it still involves a great deal of laboratory work as tens or hundreds of images need to be counted for all of the coding nanoparticles.

**Figure 1:**
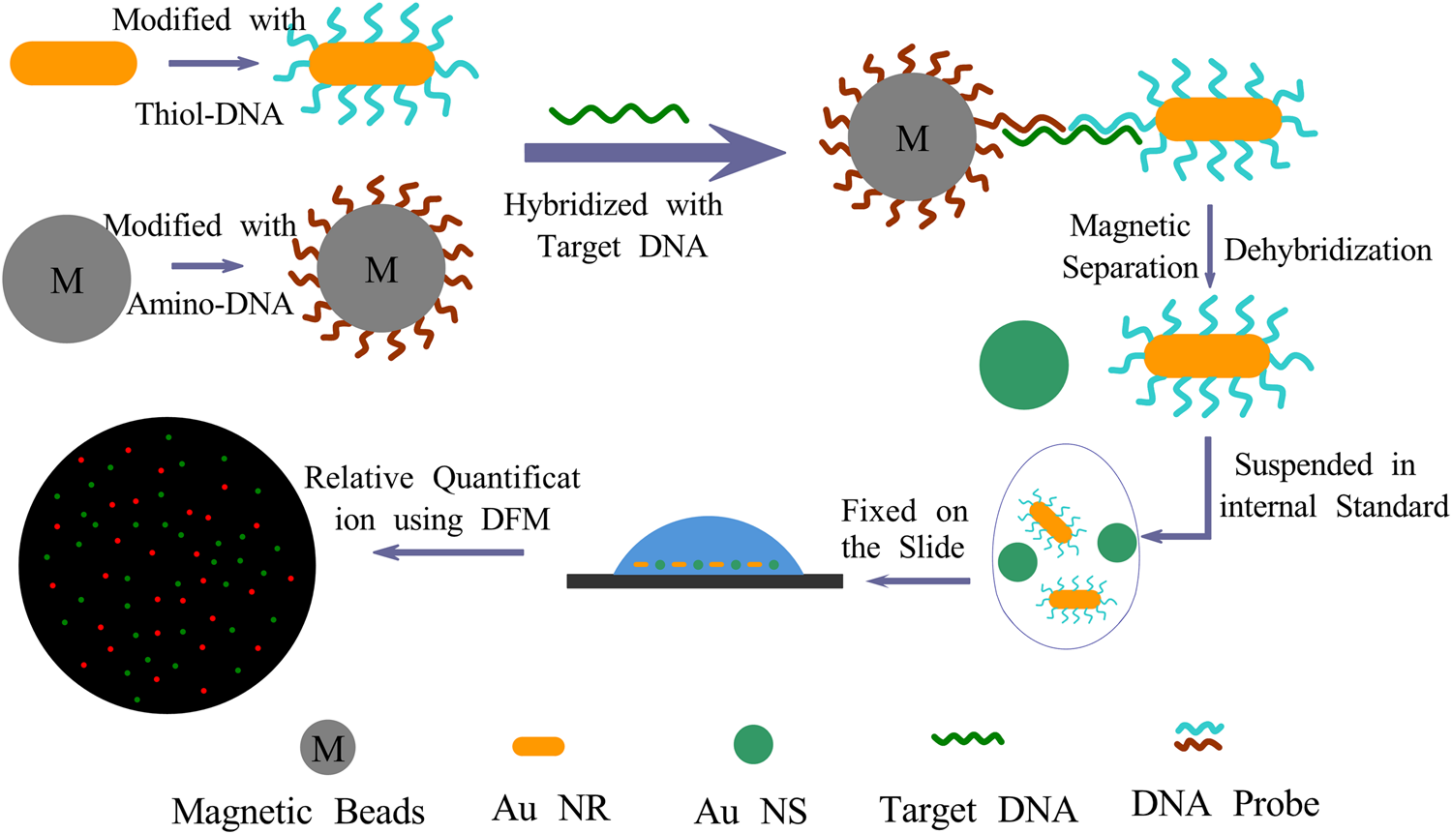
Schematic illustration for the detection of the target DNA.

Herein, we report a method to facilitate rapid and highly sensitive detection of DNA based on the coding of single target DNA with a single-countable nanocrystal and detection of the relative number ratio of the coding nanocrystal to the internal standard nanocrystal at a certain concentration. This process is illustrated in Fig. 1. First, gold nanorods (AuNRs) and magnetic beads (MBs) were modified with DNA probes called coding-AuNRs and MB probes, which were each complementary to one-half the target DNA. Mixing the MBs and target solution allowed hybridization of the DNA probes on the MBs to the other half of the target DNA molecules. Subsequent addition of the coding-AuNRs resulted in a sandwich structure, MBs-DNA-AuNRs, and the encoding of every DNA target molecule with a microscopically countable nanoparticle. Therefore, the concentration of target DNA is proportional to the number of coding-AuNRs. Upon magnetic separation, free AuNRs were thoroughly removed. Following de-hybridization, the coding-AuNRs that dissociated from the surface of the MBs were re-suspended in a buffer containing 11-mercaptoundecanoicacid (MUA) functionalized Au nanospheres (AuNSs) at a certain concentration as an internal standard. In this procedure, a large excess of coding-AuNRs were applied to reduce the possibility of multiple attachments of the target DNA to a single AuNR. A positively charged glass slide modified with an amino-terminated self-assembled monolayer was used to immobilize the negatively charged coding-AuNRs and internal standard AuNSs to overcome Brownian motion through electrostatic interactions, which allowed us to detect the number ratio of the two types of nanocrystals by a dark field microscopy (DFM) (Fig. S1). The concentration of the target DNA, which is in proportional to the coding-AuNRs, was obtained from the number ratio and known concentration of the internal standard nanocrystal.

## Methods

### Materials

Magnetic beads functionalized with carboxylic acid groups (2.7 μm in diameter) were purchased from Thermo Fisher Scientific (USA). 3-Aminopropyltriethoxysilane (APTES) was purchased form Nanjing Chen Gong organic silicon material co., LTD (China). 1-Ethyl-3-(3-dimethylaminopropyl) carbodiimide hydrochloride (EDC) was purchased from the Tokyo Kasei Kogyo Co. (Japan), and the EDC solutions used in this work were made immediately prior to use. Sodium-dodecyl sulfate (SDS), Tris-(2-carboxyethyl)-phosphine (TCEP), hydrogen tetrachloroaurate (III) hydrate (HAuCl_4_·3H_2_O), 2-(N-Morpholino) ethanesulfonic acid (MES), ethanolamine, sodium borohydride (NaBH_4_), cetyltrimethyl ammonium bromide (CTAB), silver nitrate (AgNO_3_), polyvinylpyrrolidone (PVP, M.W. 8,000), and ascorbic acid were purchased from Sigma-Aldrich. All of the DNA sequences were purchased from Beijing Genomics Institution (China). For all experiments, ultrapure water was used.

### Synthesis of AuNSs

Here a reported seeded growth approach was used to obtain the AuNSs^13^. To synthesize AuNSs, 18 nm AuNSs seeds were obtained firstly. In brief, 100 mL of 0.24 mM HClO_4_ aqueous solution was added to a 250-mL round-bottom flask and heated to 110 °C and kept for 5 min. Then 10 mL of 14.55 mM sodium citrate solution was injected quickly and stirred vigorously for 20 min. The resulting solution was placed in an ice bath to cool it to stop the seeds growing. The cooling solution was filtered by a 0.22-μm filter. The filtered AuNSs were centrifuged at 8000 rpm for 15 min to remove the smaller AuNSs and then centrifuged at 4000 rpm for 10 min to remove the larger AuNSs. In this procedure, the volume of the solution remained unchanged. As a result, monodispersed seeds with average diameter of 18 nm were finally prepared. Secondly, the obtained seeds growed larger. 2.2 mL of AuNS seed solution and 20 mL of ultrapure water were added into a 50-mL round-bottom flask and mixed well. One milliliter of 40 mM NH_2_OH·HCl solution and 1 mL of 3.52 mM HAuCl_4_ solution were slowly added drop by drop alternately. After the adding process, 5 mL of 0.3% SDS solution was added to prevent the aggregation of the grown AuNSs.

### Synthesis of AuNRs

AuNRs were synthesized according to a reported seed mediated method^14^. First, the seed solution was prepared. Forty microliters of 24 mM HAuCl_4_ and 4 mL of a 100 mM CTAB solution were mixed in a 20-mL round-bottom flask with vigorous shaking. Then, 24 μL of a 0.1 M freshly prepared ice-cold NaBH_4_ solution was rapidly added to the mixture, and the reaction was left to proceed for 1.5 min at room temperature. The color of the mixture changed to yellow brown immediately, indicating formation of the seed solution. To grow the seeds into AuNRs, 28 μL of the seed solution, 20 mL of a 0.2 M CTAB solution, 50 μL of a 40 mM AgNO_3_ solution, 160 μL of 0.1 M ascorbic acid, and 150 μL of 5 M HCl were mixed thoroughly, and the mixture was left to stand at room temperature for 12 h until the color turned blue, indicating the formation of AuNRs. The resulting solution was centrifuged for 10 min at 8600 rpm, the supernatant was removed, and the sediment re-dispersed into 10 mL of a 0.01 M CTAB solution.

### Preparation of coding-AuNR probes

The preparation of coding-AuNR probes means loading thiol-DNA (5′-SH-A AA AAA AAA TAC CAC ATC ATC CAT-3′) onto AuNRs. This process was mainly guided by previously reported work^15^ with several modifications. In this work, synthesized AuNRs were covered with CTAB molecules. A mixture of PVP and sodium dodecyl sulfate (SDS) was used to exchange the CTAB molecules. The obtained AuNRs were easily displaced by thiol-functionalized molecules, such as thiol-DNA. AuNRs were suspended in a 0.01 M pH 8.0 phosphate buffered saline (PBS) solution and centrifuged for 15 min at 9000 rpm twice to remove excess CTAB. The resulting AuNRs were re-suspended in 10 mL of 0.01 M PBS. The obtained AuNR suspension and 10% (w/v) PVP solution in ethanol were mixed well in a round-bottom flask. This mixture was stirred overnight at 40 °C. After stirring, the CTAB capped on the AuNRs was gradually exchanged using a mixture of PVP and SDS. The mixture was centrifuged for 15 min at 9000 rpm and was re-suspended in 0.01 M PBS containing 0.03% SDS three times. The resulting AuNRs were re-suspended in 1 mL of 0.01 M PBS. Then, 200 μL of a 3 mM TCEP protected thiol-DNA solution in PBS buffer was added to the post-exchanged AuNR suspension. This AuNR suspension was sonicated for 10 s before it was left to stand for 20 h. One-hundred-twenty microliters of a salting solution containing 0.01 M PBS, 0.3 M NaCl, 4 mM MgCl_2_, and 0.03% SDS was added. This salting process was repeated 10 times with a time interval of 0.5 h. After addition of the last salting solution, the solution was left to stand at room temperature for another 12 h.

### The preparation of the MBs probes

After preparing the AuNR probes, magnetic beads (MBs) with carboxyl groups on the surface were modified with amino-DNA to prepare MB probes. The basic process used to conjugate capture probes onto MBs was predominantly based on the manufacturer’s instructions. EDC was used to form an amide bond between the amino-DNA and carboxyl-MBs. One-hundred microliters of a 20 mg/mL MB solution was washed with a 50 mM MES pH 5.0 buffer containing 0.03% SDS. After two washes, 100 μL of the 50 mM MES buffer was added and mixed. Seventy microliters of 100 μM amino-DNA (5′-ATA ACT GAA AGC CAA AAA AAA AAA A-NH2-3′) in MES buffer was added and mixed using a vortex mixer. Fifty microliters of a 50 mg/mL freshly prepared EDC solution in ice-cold MES buffer was added and mixed well using a vortex mixer before being incubated for 30 min at room temperature with a slow tilt rotation. After which, the MBs were collected by application of a magnet and the supernatant was pipetted off to quantify the conjugated amino-DNA. The coated MBs were incubated in 50 μL of 50 mM ethanolamine in PBS pH 8.0 for 60 min at room temperature with a slow tilt rotation to quench the unreacted activated carboxylic acid groups on the MBs. The MBs were collected and washed 4 times with 200 μL of 50 mM Tris buffer to remove excess amino-DNA.

### The detection of the target DNA

The target DNA molecules were then mixed with the MB and AuNR probes successively to obtain a sandwiched structure between the MBs and AuNRs. In this work, the target oligonucleotide associated with the hepatitis B virus (HBV) was chosen as a model system to demonstrate the applicability of the current assay for highly sensitive and typical DNA detection. In a typical experiment, 20 μL of the MB probe with a total number of ~4 × 10^7^ was added to a 2-mL micro-centrifuge tube. Tw-hundred microliters of the target DNA (5′-TTG GCT TTC AGT TAT ATG GAT GAT GTG GTA-3′) test sample solution containing 0.01 M PBS buffer, 0.01% SDS, and 0.15 M NaCl was added. The hybridization reaction between the target molecules and capture probes on the MB surface was conducted, with slow tilt rotation at 37 °C for 1 h. A magnet was introduced to separate the MBs. After the washing procedure, the MBs were re-suspended in 50 μL of a solution containing 0.01 M PBS, 0.15 M NaCl, and 0.01% SDS. Subsequently, 200 μL of a AuNR probe solution with a total number of ~7 × 10^9^ was added. AuNR codes were hybridized with target molecules that were bound to the MBs with a slow tilt rotation at 37 °C for 1 h. A magnet was used to separate the MBs. The supernatant was removed, and the MBs were washed with a washing buffer (0.01 M PBS, 0.15 M NaCl, and 0.1% SDS) 5 times to remove all of the unreacted AuNRs. After the last washing procedure, the aggregated MBs were re-suspended in 10 μL of a standard 11-mercaptoundecanoicacid (MUA) solution that contained gold nanospheres (AuNSs) at a certain concentration before being transferred into a 200-μL clean tube. The tube was placed into a 60 °C water bath for 10 min to de-hybridize the double stranded DNA. The MBs were collected in the bottom of the tube using a strong magnet. Three microliters of the supernatant was dropped onto a clean and dry APTES-modified slide. Then, the slide was covered with a clean, dry coverslip to prepare the microscope specimen for dark field imaging.

### The modification of the glass slide

AuNRs and AuNSs are very small in solution, such that there is fierce Brownian motion, which affects imaging, leading to incorrect counting. In this work, AuNSs and AuNRs were modified with a MUA solution, and the slides used were modified by 3-aminopropyltriethoxysilane (APTES). The glass slides were first immersed in a piranha solution (30% H_2_O_2_:98% H_2_SO_4_ = 3:7) for 6 h before being sonicated in ultrapure water several times to thoroughly remove any dust. The clean slides were then dried in a vacuum oven at 60 °C for 3 h. Then, the dried glass slides were incubated in a 10% v/v APTES ethanol solution for 12 h before being extensively rinsed in ethanol with sonication 4 times. The slides were then dried in a vacuum oven at 60 °C for 6 h. In this way, large amounts of amino groups were successfully incorporated onto the surface of the glass slides, which combined with the carboxylic acid groups on the surface of the AuNSs and AuNRs.

### Dark field microscopic imaging of Au NRs and NSs

An upright optical microscope (BX53, Olympus) equipped with a NA = 1.20–1.43 oil immersion dark field condenser was used for imaging. The white light source used was a 150 W halogen lamp. A 40 × objective was used to collect the scattered light from the AuNRs and AuNSs. A color CCD camera (DP73, Olympus, 1600 × 1200 pixels) with an integration time of 500 ms was used. The size of the image obtained was approximately 0.34 × 0.26 mm^2^, and the size of the coverslip was 22 × 22 mm^2^. This means that approximately 8003 images were obtained for every sample.

### Classifying and counting multicolor dots

In this work a homemade batch-to-batch Matlab program was developed to accurately and automatically classify and count the multicolor dots in an image. Then we will introduce the main idea of the preparation of the program. Firstly, read in image information and binaries them and tab the connective region. One colored dot in the DFM image corresponds to a connective region. Then extract the hue value of each pixel and judge which hue range the pixel is. According the hue range of most pixels in the connected domain determine the color of the dot and count it. If all of the RGB values are greater than the thresholds, regard them as white pixels. If the number of white pixels is greater than the thresholds, regard them as the contaminants, and eliminate them. The detailed Matlab program shows in the supporting information.

## Results and Discussion

Due to the localized surface plasmon resonance (LSPR) effect^16–18^, individual noble metal nanocrystals scatter light strongly with a distinct color, allowing it to be clearly distinguished by a DFM^19,20^. Remarkably, AuNRs and AuNSs can be easily distinguished by a non-scanning DFM owing to the different color of light that they scatter^21,22^. To reduce the labor intensity and errors due to the subjective judgement of colors in practical applications, a homemade batch-to-batch Matlab program was developed to accurately and automatically classify multicolor dots in an image. The CIE (Commission Internationale Ed I’eclairage) color space^23,24^, a mathematic abstraction of the color feature, is used as a unified criterion for the AuNRs and AuNSs and to remove the potentially contaminating particles. The chromaticity coordinates of the colored dots in the DFM image were calculated by Equations (1) and (2)^25,26^, where I_R_, I_G_, and I_B_ are the intensity of the three components of the RGB values obtained from an 8-bit color CCD camera.

1 $$(\begin{array}{c}X\\ Y\\ Z\end{array})=\frac{1}{0.17697}(\begin{array}{ccc}0.49 & 0.31 & 0.20\\ 0.17697 & 0.81240 & 0.01063\\ 0 & 0.01 & 0.99\end{array})(\begin{array}{c}IR\\ IG\\ IB\end{array})$$

2 $$(\begin{array}{c}x\\ y\\ z\end{array})=\frac{1}{X+Y+Z}(\begin{array}{c}X\\ Y\\ Z\end{array})$$

**Figure 2:**
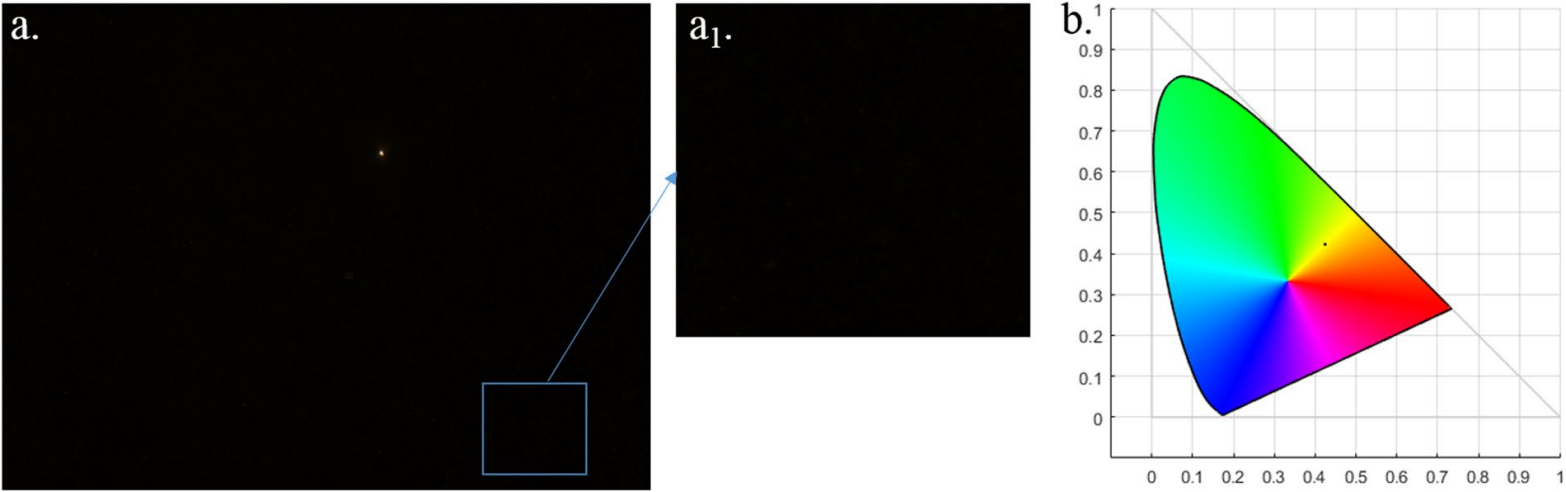
(**a**) Typical DFM image of the ultrapure water sample. Image (**a**_**1**_) is the partial enlarged views of (**a**). Image (**b**) is the chromaticity distribution of the potential contamination particles in DFM image (**a**), as shown in the CIE color space.

**Figure 3:**
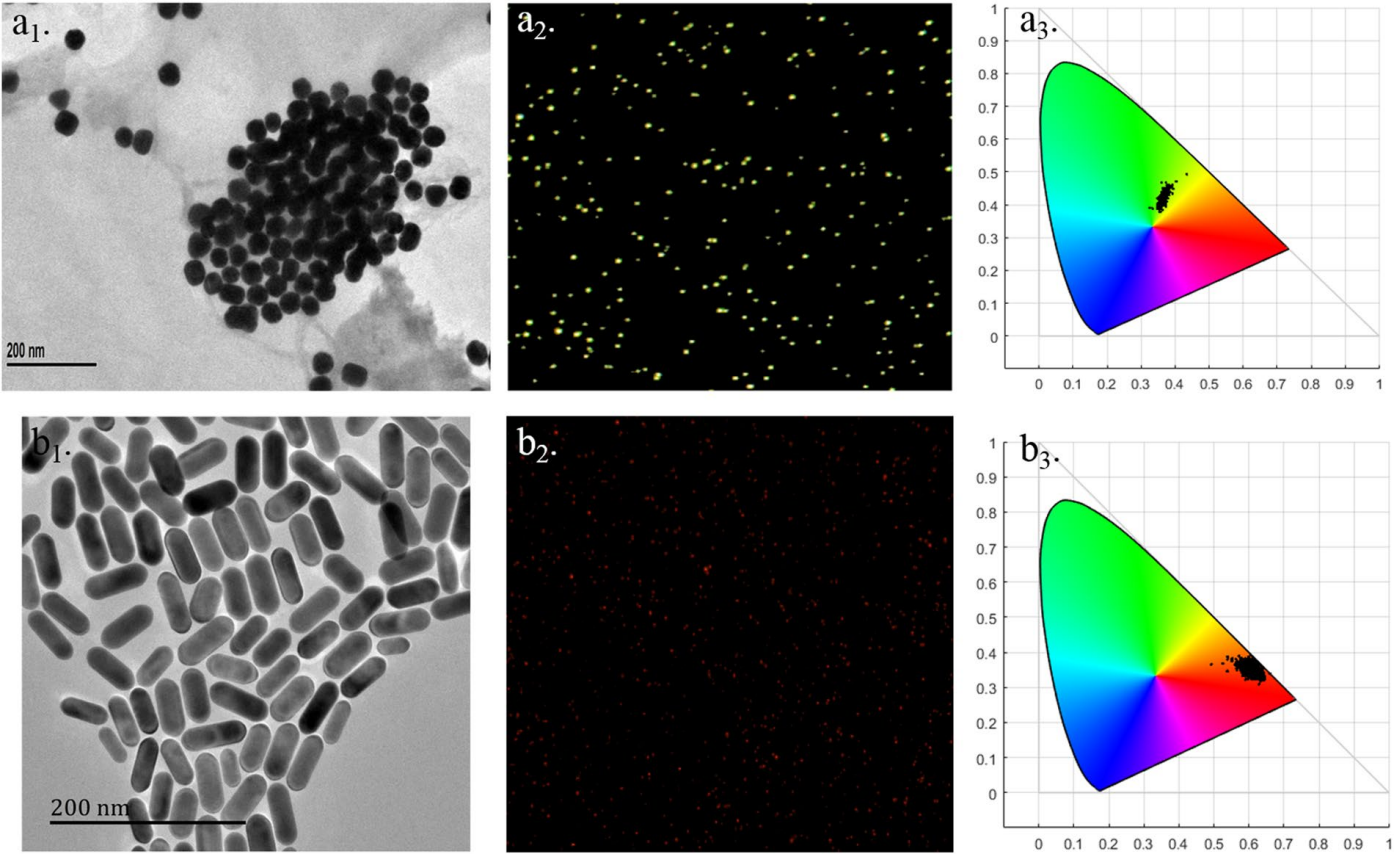
A typical TEM image of the as-synthesized AuNSs (**a**_**1**_) and AuNRs (**b**_**1**_). A typical DFM image of the AuNSs (**a**_**2**_) and AuNRs (**b**_**2**_). The representative chromaticity distribution of the typical Au NSs (**a**_**3**_) and Au NRs (**b**_**3**_) in the CIE color space.

Figure 2 shows a DFM image of an ultrapure water sample on a glass slide with a few contamination particles, which can be eliminated by the homemade Matlab program. In this work, the average diameter of the gold nanospheres (AuNSs) (as shown in Fig. 3(a_1_)) that were used as an internal standard is 49 nm, as obtained from a laser particle size analyzer^13^, and the extinction peak is at 531 nm (shown in Fig. S2(a)). The Au NRs are rod-like structures^27^, as shown in Fig. 3(b1), with an average length and diameter of 53 and 25 nm, respectively. The extinction peaks located at 515 and 659 nm (shown in Fig. S2(b)) correspond to the transverse and longitudinal plasmonic modes, respectively^28^. The significant differences in the spectrum features can be clearly reflected in the colored DFM images, as shown in Fig. 3(a2,b2) ^15^. Figure 3(a3) and (b3) shows the representative chromaticity distribution of the AuNSs and AuNRs in the CIE color space. From the CIE chromaticity diagram, it is clear that large quantities of AuNSs are concentrated in the green-colored region, which is different from the red-colored region, which represents AuNRs. It is evident that the AuNSs and AuNRs can be distinguished using their color features and that the crosstalk between them is very low. Some spots scatter for the region, perhaps due to contamination particles or AuNSs/AuNRs with significant size differences. Because the AuNSs and AuNRs show a different LSPR peak wavelength, and hence a different scattering color, it is reasonable to use AuNSs at a certain concentration as an internal standard nanocrystal for the enumeration of the coding AuNR.

**Figure 4:**
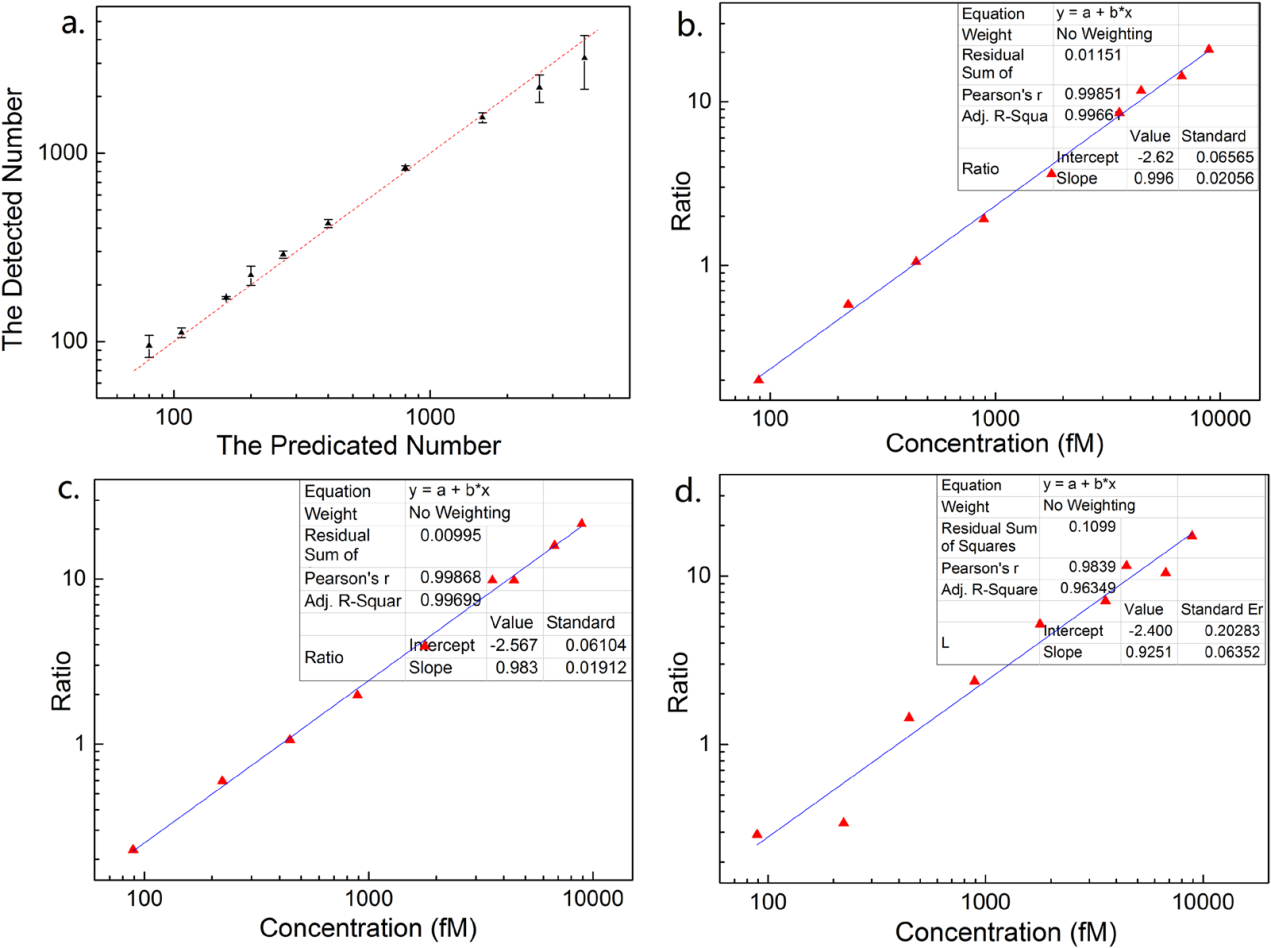
Graph (**a**) shows the changes of the average recorded number of green dots in every DFM image with the changes of the predicated number, and the red dotted line represents the function of y = x. Graphs (**b**,**c** and **d**) show the changes in the number ratio of the red dots to the green dots in every DFM image when 20, 10, and 5 images are used to comprise a data point, respectively, and the blue lines is the linear fit.

In our previous work^12^, it has been demonstrated that the concentration of nanocrystals can be obtained by counting the number of colored dots in the DFM image, and the quantitative accuracy is dominated by Poisson statistics. To confirm the concentration of AuNSs used as the internal standard, the mass of gold in 1 mL of the synthetic AuNS solution was determined to be 16.54 ppm by using inductively coupled plasma atomic emission spectrometry (ICP AES). Given that the mass of every nanosphere is 1.19 × 10^−12^ mg^29^, the concentration of AuNSs was calculated to be 23.05 pM. Figure 4(a) shows the changes of the average detected number of green dots in every DFM image with the changes of the predicated number, which indicates that the detected numbers are very close to the predicted ones, from tens up to a thousands of dots per frame. In most cases, the detected number is slightly smaller than the predicated one, which may result from the agglomeration of AuNSs. When two or more AuNSs become much closer, only one dot with a yellow or orange color will be recorded in the image, and after processing the image using the Matlab program, these dots are removed. When the concentration is 0.45 pM, the number of green dots in every frame is approximately 100. In addition, the quantity of AuNSs obtained through dark-field imaging matched the calculated number from the inductively coupled plasma atomic emission spectroscopy (ICP AES) very well, indicating that the agglomeration had a smaller effect on the accuracy less. Therefore, AuNS with a concentration of 0.45 pM was used as the internal standard nanospheres (ISNSs).

Owing to the homogeneous dispersion properties, the AuNRs and AuNSs are evenly distributed over the specific region of a surface. Typically the number ratio of red to green dots in different DFM images taken from the same specimen do not greatly differ even though the difference in absolute numbers may be significant. It is therefore possible to obtain the concentration of AuNRs by recording the relative number ratio of AuNRs to ISNSs. Figure 4(b) shows the changes of the AuNRs to ISNSs ratio with the AuNR concentration when 20 images are taken from one specimen to comprise one data point. Figure S3 shows the exact location from which the images are taken, and 4 images were taken from each small grey square. The entire volume of the test solution is 3 μL. From the graph, we can see that the ratio of AuNRs to ISNSs changes linearly with the concentration of AuNRs. Pearson’s r is 0.99851, which means that the linearity is good. From the number ratio of the AuNRs to the ISNS, the concentration of the AuNRs was calculated to be 70.62 pM, which is very close to the result obtained from the absolute counting, 67.79 pM, as obtained from Fig. S4(a), with a variation less than 5%. It is evident that the method using AuNSs as internal standards works well. The acquired images were further reduced to 10 frames from the same specimen with 2 images from each grey square, as illustrated in Fig. S3. Figure 4(c) shows the changes of the number ratio with the changes in the concentration of AuNRs when 10 images were acquired. Pearson’s r is 0.99868, which means that the correlation between the ratio and concentration of AuNRs is linear. From the ratio, we can calculate that the average concentration is 73.68 pM, which is still close to that obtained by absolute counting with a variation of 8.7%. Figure 4(d) and Fig. S4(b) show that when 5 (1 image was taken from every grey square) and 3 images (1 image was taken randomly from three grey squares among the five) were used to comprise a data point, the number ratio changed with the AuNR concentration. Pearson’s r is 0.9839 and 0.9788, and the calculated concentration is 73.38 and 59.07 pM, with a variation of 8.2 and 12.9%, respectively. Although the counting accuracy is compromised compared with that using the absolute counting method, it should be noted that for the absolute counting of coding AuNRs enriched in an area as small as a few square millimeters, a few hundred images need to be recorded. In addition, the alignment of acquired images takes a great amount of time and effort. Any overlap and gaps between adjacent images may result in an inaccurate final number. By using ISNS, it is clear that data generated from 5 images from evenly designated regions are adequate due to the homogenous nature of the nanoparticles and allow one to save more time and apply the method more rapidly.

**Figure 5:**
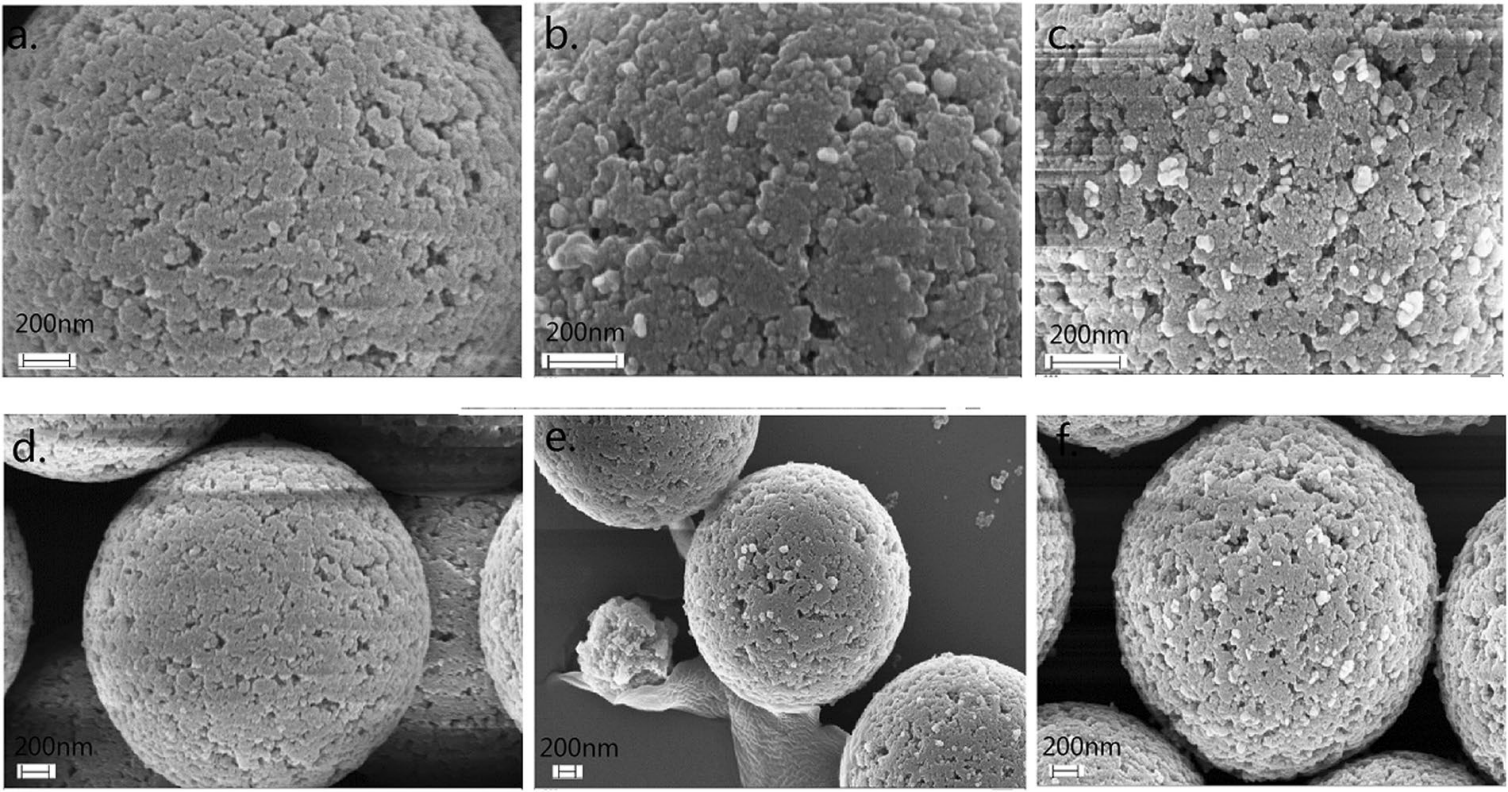
Typical SEM images of magnetic beads loaded with AuNRs by the aid of DNA hybridization between complementary DNA. The concentration of target DNA used was (**a**) 0, (**c**) 1, and (**e**) 4 pM, respectively. (**b**) 0, (**d**) 1, and (**f**) 4 pM are the corresponding whole images of the magnetic beads.

**Figure 6:**
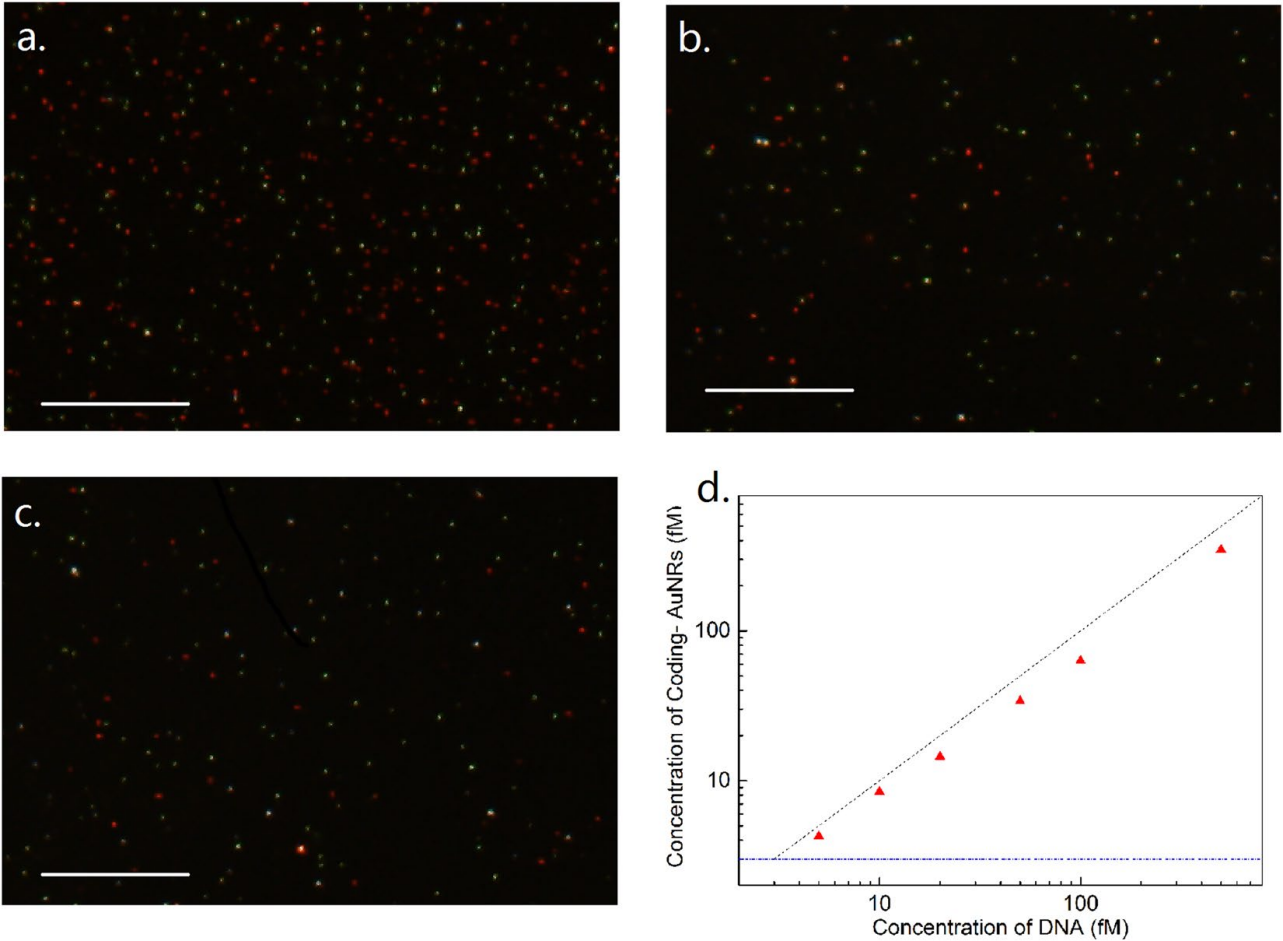
(**a**,**b** and **c**) show typical DFM images of the coding-AuNRs and the ISNSs when the concentration of target DNA is 100, 50, and 20 fM, respectively, and the number ratio is approximately 3.0, 1.5, and 0.6. The scale bar is 20 µm. Graph (**d**) shows the changes of the concentration of the coding-AuNRs with that of the concentration of the target DNA. The black dash dotted line shows the function y = x, and the blue line indicates the background signal.

The above approach for the quantification of AuNRs was then used to develop a new method for the highly sensitive and rapid detection of DNA by encoding target DNA with AuNRs. To be more accurate (the Pearson’s r should be larger than 0.99), 10 images were recorded to comprise a data point. As shown in Fig. 1, the AuNRs were first coupled with thiol-DNA probe**s** (5′-SH-A AA AAA AAA TAC CAC ATC ATC CAT-3′) to prepare coding-AuNRs, and the surface potential measurement was used to confirm that the DNA probes capped the AuNRs. CTAB-capped AuNRs had a zeta potential of + 30.7 mV, while a negative zeta potential of −39.4 mV was observed after the DNA modification process. Then, the carboxyl group modified magnetic beads (MBs) coupled with an amino-DNA probe (5′-ATA ACT GAA AGC CAA ATG GAT GAT GTG GTA-3′) was chosen as a model system to demonstrate the applicability of the internal standard method. Half of the hepatitis B virus (HBV) associated DNA was complementary with the DNA probe on the AuNRs, and the other half was complementary with the DNA probe on the MBs. The target DNA was first captured by the MBs, followed by labelling with coding-AuNRs to obtain the sandwich structure of MBs-DNA-AuNRs by DNA hybridization. In this way, the detection of target DNA is therefore transferred to the detection of coding AuNRs because almost every single target DNA is capped by a single AuNR, and the concentration of target DNA is proportional to that of coding-AuNRs. Then a magnet was used to collect the MBs, and the supernatant removed. The MBs were then washed several times to remove all free AuNRs. After the last washing procedure, the aggregated MBs were re-suspended in a MUA solution of ISNSs. The coding-AuNRs were released from the sandwich structure through thermal de-hybridization of the double stranded DNA. Subsequently, a positively charged glass slide modified with an amino-terminated self-assembled monolayer was used to immobilize the negatively charged coding-AuNRs and the ISNSs for DFM measurement. Then, the number ratio of the coding-AuNRs to the ISNSs was obtained and the concentration of the coding-AuNRs, which is proportional to the target DNA, was calculated. The success of the two-step hybridization procedure was qualitatively evaluated by the ability to form the sandwich structure. Figure 5 shows a typical SEM image of magnetic beads loaded with AuNRs via DNA hybridization between complementary DNA. In the control experiment where no target DNA was present, as shown in Fig. 5(a,d), few AuNRs were observed on a magnetic bead surface. However, when the concentration of the present target DNA was 1 and 4 pM, as shown in Fig. 5(b)/(e) and (c)/(f), we observed that large quantities of AuNRs were attached on the surface of the magnetic beads; and the more target DNA, the more AuNRs were captured. Figure 6(a,b and c) show typical DFM images of the coding-AuNRs and the ISNSs when the concentration of the target DNA was 100, 50, and 20 fM, respectively. From Fig. 6(a,b and c), we observed that the more target DNA was added, the larger the ratio of red to green dots. Figure 6(d) shows the concentration of the coding-AuNRs calculated from the number ratio as a function of the concentration of the target DNA. In this experiment, after the washing process, the MBs were re-suspended in 10 μL of ISNSs, and the volume of the target DNA under detection was 200 μL. This indicates the detected coding-AuNRs were 20 times concentrated and were equivalent to the use of 22.5 fM rather than 0.45 pM ISNS for the calculation of the target molecule concentration. The obtained data points had a nearly linear curve within the concentration range. Six different concentrations of the target DNA of 500, 100, 50, 20, 10, and 5 fM were tested, and the results demonstrated that this method works well. Each data point of the curve was generated from 10 images. The black dashed dotted line in Fig. 6(d) indicates the function of y = x, and all of the data points in the graph are below it, which means that the efficiency was lower than 100%. This is because not all of the target DNA could be captured in the sandwich structure, and some of the sandwich structure may be destroyed during the washing process. The detection limit of an analytical assay depends of the sensitivity and noise level of the method. Digital detection allows ultrahigh sensitivity of the system. However, the noise level over the whole slide was significantly increased compared to that if a very small area was employed. The limit of detection reported using the current method was ~3 fM, which was due to the background signal, as shown by the blue dashed dotted line in Fig. 6(d). For comparison, the detection sensitivity of the method developed here does not differ considerably compared with that of the digital ELISA^30^ or the methods based on the single QD nanosensor^31^. As for label-free methods, such as SPR^32^ and micro-balance^33^, could be indeed more convenient and rapid, but it generally suffers low sensitivity and high detection limit. In this method, the nanoparticles synthesized and probes prepared at one time can be used for hundreds of tests and the probes can be stored for at least a few months before use. This will reduce the whole testing time significantly. On the other hand, this methodology offers high sensitivity with limit of detection down to low femto-molar level, which is superior to label-free approach. In the experiment, nearly ~70% of the target DNA was encoded by the AuNRs and separated from the sandwich structure. The ability to achieve rapid and high detection sensitivity in this method depends on accurately obtaining the number ratio of coding-AuNRs to ISNSs because of the large scattering section and their distinct color features. At the same time, efficient coding of the target molecules contributes to the advantages of this method. Finally, the low background noise of this method also plays a very important role in achieving accurate and rapid results.

## Conclusion

In conclusion, microscopic differentiation and enumeration of nanoparticles enables recording of the number ratio of AuNRs to AuNSs, which allows the concentration of the AuNRs to be obtained rapidly and accurately. By encoding target DNA with these AuNRs, a simple and robust method for the digital detection of target biomolecules is achieved. The method reported here offers a possible approach to rapidly detect clinically significant DNA at low femto-molar levels with reasonable accuracy. Additionally, it is promising to generalize the current method to multiplexed detection by using different nanocrystals to encode different types of target DNA, paving the way for wide-spread practical applications^34^.

## Electronic supplementary material

Supplementary information
