# Supplementary material for: Microscopic Differentiation of Plasmonic Nanoparticles for the Ratiometric Read-out of Target DNA
Source: Sci Rep. 2017 Nov 7;7:14742. doi: 10.1038/s41598-017-15256-1 (PMC5677009; doi:10.1038/s41598-017-15256-1)
Supplement: Supplementary file 1 — Supplementary information [file 41598_2017_15256_MOESM1_ESM.pdf]

Microscopic Differentiation of Plasmonic Nanoparticles for Ratiometric Read-out of Target DNA

Zhenjie Wu,[1,2] Rui Yang,[1] Di Zu,[1,2] and Shuqing Sun*[1,2]

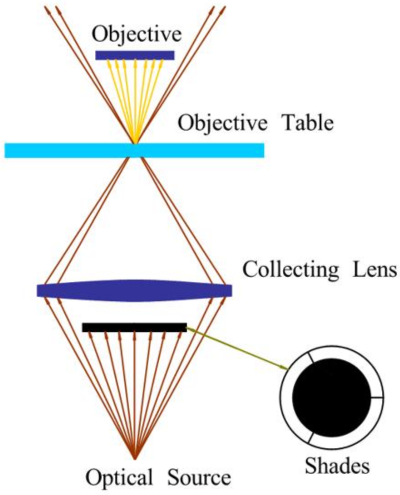

Figure S1. Schematically illustration for a typical dark field microscope.

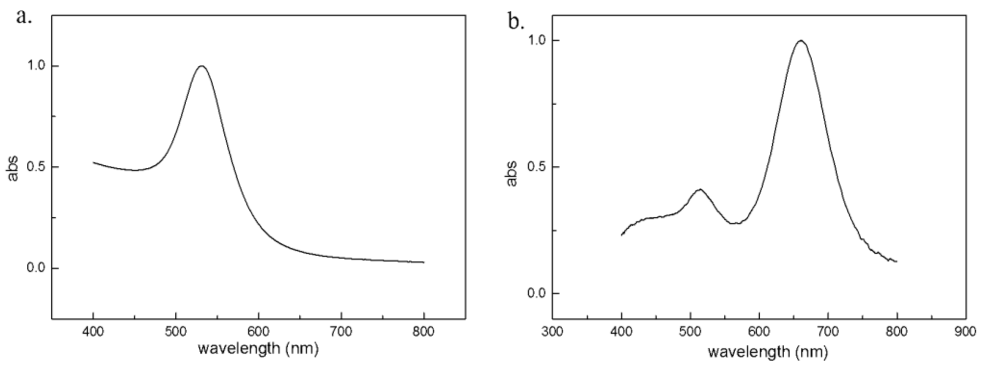

Figure S2. The normalized extinction spectra of the synthetic (a) AuNSs and (b) AuNRs

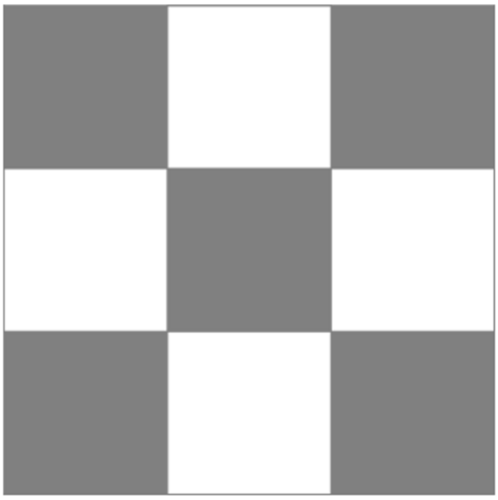

Figure S3. The exact location where we take the images. The square means the coverslip and the small ones filled with gray are the locations where we take images.

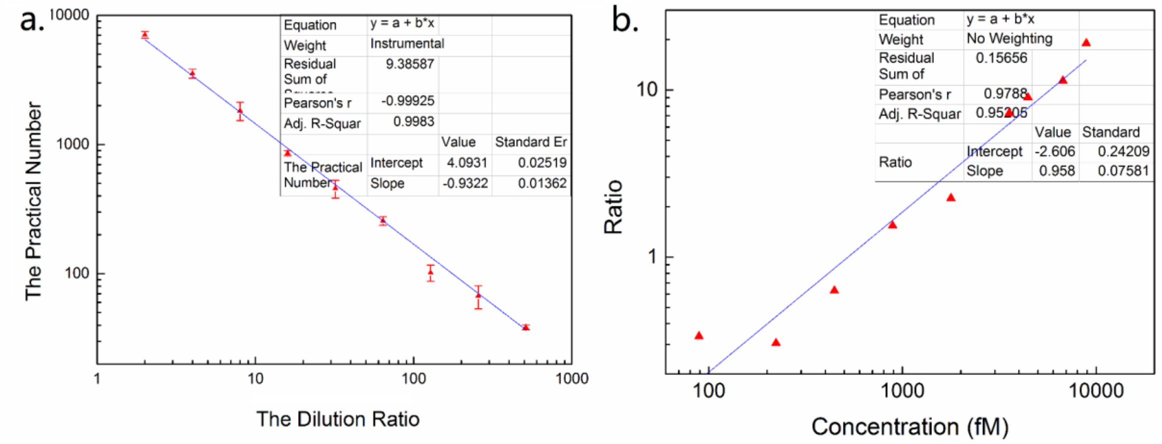

Figure S4. Graph (a) shows the number of the AuNRs in every DFM image by absolutely counting the number of the red dots. Graph (b) shows the number ratio of the red dots to the green one in every DFM image when 3 images were used to comprise a data point. The blue dot line means the result of the linear fitting.

Detailed Matlab program to classify and count multicolor dots.

```
clc;
clear;
close all;
oriI = imread('G:\MyWork\Third\Manuscript for APL\used as figure\figure5\1000\1.tif');
desir = [0.07,0.5,0.9];
wthresh = 20;
wpercent = 0.20;
I = oriI(:,:,1:3);
I1 = oriI(:,:,1);
```

```
%I2 = oriI(:,:,2);
%I3 = oriI(:,:,3);
figure
imshow(I);
level = graythresh(I);
bwI = im2bw(I,2*level);
figure
imshow(bwI)
[label, numObj] = bwlabel(bwI,4);
K = rgb2hsv(I);
centerObj = zeros(numObj,3);
for i = 1:numObj
    [x,y] = find(label==i);
    xmean = floor(mean(x));
    ymean = floor(mean(y));
    centerObj(i,:) = K(xmean,ymean,:);
end
figure
scatter(centerObj(:,1),centerObj(:,2))
bwAim = zeros([size(bwI),length(desir)]);
  numAim = zeros(1,length(desir));
for i = 1:numObj
    [x,y] = find(label == i);
    numall = length(x);
        numdesir = zeros(1,length(x));
    numwhite = 0;
    for j = 1:numall
        if min(I(x(j),y(j),:))>= wthresh
            numwhite = numwhite + 1;
        end
        if K(x(j),y(j),1)>desir(end)
            numdesir(j) = 1;
        else
            numdesir(j) = find(desir>=K(x(j),y(j),1),1);
        end
    end
    if numwhite/numall >= wpercent
        continue;
    end
    alldesir = zeros(1,length(desir));
    for j = 1:length(desir)
        alldesir(j) = length(find(numdesir==j));
    end
    maindesir = find(alldesir >= (numall/length(desir)));
```

```
        if length(maindesir)==1
            for j = 1:numall
                bwAim(x(j),y(j),maindesir(1)) = 1;
            end
            numAim(maindesir(1)) = numAim(maindesir(1)) + 1;
        elseif length(maindesir) > 1
            for j = 1:length(maindesir)
                aimdesir = find(numdesir == maindesir(j));
                for k = 1:length(aimdesir)
                    bwAim(x(aimdesir(k)),y(aimdesir(k)),maindesir(j)) = 1;
                end
                numAim(maindesir(j)) = numAim(maindesir(j)) + 1;
            end
        end
    end
    for i = 1:length(desir)
        nI = zeros(size(I));
        nI(:,:,1) = double(I(:,:,1)).*bwAim(:,:,i);
        nI(:,:,2) = double(I(:,:,2)).*bwAim(:,:,i);
        nI(:,:,3) = double(I(:,:,3)).*bwAim(:,:,i);
        figure
        imshow(uint8(nI))
    end
```
